# Language impairment in the genetic forms of behavioural variant frontotemporal dementia

**DOI:** 10.1007/s00415-022-11512-1

**Published:** 2022-12-20

**Authors:** Kiran Samra, Amy M. MacDougall, Arabella Bouzigues, Martina Bocchetta, David M. Cash, Caroline V. Greaves, Rhian S. Convery, John C. van Swieten, Harro Seelaar, Lize Jiskoot, Fermin Moreno, Raquel Sanchez-Valle, Robert Laforce, Caroline Graff, Mario Masellis, Maria Carmela Tartaglia, James B. Rowe, Barbara Borroni, Elizabeth Finger, Matthis Synofzik, Daniela Galimberti, Rik Vandenberghe, Alexandre de Mendonça, Christopher R. Butler, Alexander Gerhard, Simon Ducharme, Isabelle Le Ber, Pietro Tiraboschi, Isabel Santana, Florence Pasquier, Johannes Levin, Markus Otto, Sandro Sorbi, Jonathan D. Rohrer, Lucy L. Russell, On Behalf of the Genetic FTD Initiative (GENFI), Annabel Nelson, David L. Thomas, Emily Todd, Hanya Benotmane, Jennifer Nicholas, Rachelle Shafei, Carolyn Timberlake, Thomas Cope, Timothy Rittman, Alberto Benussi, Enrico Premi, Roberto Gasparotti, Silvana Archetti, Stefano Gazzina, Valentina Cantoni, Andrea Arighi, Chiara Fenoglio, Elio Scarpini, Giorgio Fumagalli, Vittoria Borracci, Giacomina Rossi, Giorgio Giaccone, Giuseppe Di Fede, Paola Caroppo, Pietro Tiraboschi, Sara Prioni, Veronica Redaelli, David Tang-Wai, Ekaterina Rogaeva, Miguel Castelo-Branco, Morris Freedman, Ron Keren, Sandra Black, Sara Mitchell, Christen Shoesmith, Robart Bartha, Rosa Rademakers, Jackie Poos, Janne M. Papma, Lucia Giannini, Rick van Minkelen, Yolande Pijnenburg, Benedetta Nacmias, Camilla Ferrari, Cristina Polito, Gemma Lombardi, Valentina Bessi, Michele Veldsman, Christin Andersson, Hakan Thonberg, Linn Öijerstedt, Vesna Jelic, Paul Thompson, Tobias Langheinrich, Albert Lladó, Anna Antonell, Jaume Olives, Mircea Balasa, Nuria Bargalló, Sergi Borrego-Ecija, Ana Verdelho, Carolina Maruta, Catarina B. Ferreira, Gabriel Miltenberger, Frederico Simões do Couto, Alazne Gabilondo, Ana Gorostidi, Jorge Villanua, Marta Cañada, Mikel Tainta, Miren Zulaica, Myriam Barandiaran, Patricia Alves, Benjamin Bender, Carlo Wilke, Lisa Graf, Annick Vogels, Mathieu Vandenbulcke, Philip Van Damme, Rose Bruffaerts, Koen Poesen, Pedro Rosa-Neto, Serge Gauthier, Agnès Camuzat, Alexis Brice, Anne Bertrand, Aurélie Funkiewiez, Daisy Rinaldi, Dario Saracino, Olivier Colliot, Sabrina Sayah, Catharina Prix, Elisabeth Wlasich, Olivia Wagemann, Sandra Loosli, Sonja Schönecker, Tobias Hoegen, Jolina Lombardi, Sarah Anderl-Straub, Adeline Rollin, Gregory Kuchcinski, Maxime Bertoux, Thibaud Lebouvier, Vincent Deramecourt, Beatriz Santiago, Diana Duro, Maria João Leitão, Maria Rosario Almeida, Miguel Tábuas-Pereira, Sónia Afonso

**Affiliations:** 1 Dementia Research Centre, Department of Neurodegenerative Disease, UCL Queen Square Institute of Neurology, Queen Square, London, WC1N 3BG UK; 2 Department of Medical Statistics, London School of Hygiene and Tropical Medicine, London, UK; 3 Department of Neurology, Erasmus Medical Centre, Rotterdam, Netherlands; 4 Cognitive Disorders Unit, Department of Neurology, Donostia Universitary Hospital, San Sebastian, Spain; 5 Neuroscience Area, Biodonostia Health Research Institute, San Sebastian, Gipuzkoa, Spain; 6 Alzheimer’s Disease and Other Cognitive Disorders Unit, Neurology Service, Hospital Clínic, Institut d’Investigacións Biomèdiques August Pi I Sunyer, University of Barcelona, Barcelona, Spain; 7 Clinique Interdisciplinaire de Mémoire, Département des Sciences Neurologiques, CHU de Québec, and Faculté de Médecine, Université Laval, Québec, QC Canada; 8 Division of Neurogeriatrics, Department of Neurobiology, Care Sciences and Society, Center for Alzheimer Research, Bioclinicum, Karolinska Institutet, Solna, Sweden; 9 Unit for Hereditary Dementias, Theme Aging, Karolinska University Hospital, Solna, Sweden; 10 Sunnybrook Health Sciences Centre, Sunnybrook Research Institute, University of Toronto, Toronto, Canada; 11 Tanz Centre for Research in Neurodegenerative Diseases, University of Toronto, Toronto, ON Canada; 12 Department of Clinical Neurosciences, University of Cambridge, Cambridge, UK; 13 Neurology Unit, Department of Clinical and Experimental Sciences, University of Brescia, Brescia, Italy; 14 Department of Clinical Neurological Sciences, University of Western Ontario, London, ON Canada; 15 Department of Neurodegenerative Diseases, Hertie-Institute for Clinical Brain Research and Center of Neurology, University of Tübingen, Tübingen, Germany; 16 Center for Neurodegenerative Diseases (DZNE), Tübingen, Germany; 17 Fondazione Ca’ Granda, IRCCS Ospedale Policlinico, Milan, Italy; 18 University of Milan, Centro Dino Ferrari, Milan, Italy; 19 Laboratory for Cognitive Neurology, Department of Neurosciences, KU Leuven, Louvain, Belgium; 20 Neurology Service, University Hospitals Leuven, Louvain, Belgium; 21 Leuven Brain Institute, KU Leuven, Louvain, Belgium; 22 Laboratory of Neurosciences, Institute of Molecular Medicine, Faculty of Medicine, University of Lisbon, Lisbon, Portugal; 23 Nuffield Department of Clinical Neurosciences, Medical Sciences Division, University of Oxford, Oxford, UK; 24 Department of Brain Sciences, Imperial College London, London, UK; 25 Division of Neuroscience and Experimental Psychology, Wolfson Molecular Imaging Centre, University of Manchester, Manchester, UK; 26 Departments of Geriatric Medicine and Nuclear Medicine, University of Duisburg-Essen, Essen, Germany; 27 Department of Psychiatry, McGill University Health Centre, McGill University, Montreal, QC Canada; 28 McConnell Brain Imaging Centre, Montreal Neurological Institute, McGill University, Montreal, QC Canada; 29 Sorbonne Université, Paris Brain Institute–Institut du Cerveau–ICM, Inserm U1127, CNRS UMR 7225, AP-HP-Hôpital Pitié-Salpêtrière, Paris, France; 30 Centre de référence des démences rares ou précoces, IM2A, Département de Neurologie, AP-HP-Hôpital Pitié-Salpêtrière, Paris, France; 31 Département de Neurologie, AP-HP-Hôpital Pitié-Salpêtrière, Paris, France; 32 Reference Network for Rare Neurological Diseases (ERN-RND), Tübingen, Germany; 33 Fondazione IRCCS Istituto Neurologico Carlo Besta, Milan, Italy; 34 University Hospital of Coimbra (HUC), Neurology Service, Faculty of Medicine, University of Coimbra, Coimbra, Portugal; 35 Center for Neuroscience and Cell Biology, Faculty of Medicine, University of Coimbra, Coimbra, Portugal; 36 Univ Lille, Lille, France; 37 Inserm 1172, Lille, France; 38 CHU, CNR-MAJ, Labex Distalz, LiCEND Lille, Lille, France; 39 Department of Neurology, Ludwig-Maximilians Universität München, Munich, Germany; 40 German Center for Neurodegenerative Diseases (DZNE), Munich, Germany; 41 Munich Cluster of Systems Neurology (SyNergy), Munich, Germany; 42 Department of Neurology, University of Ulm, Ulm, Germany; 43 Department of Neurofarba, University of Florence, Florence, Italy; 44 IRCCS Fondazione Don Carlo Gnocchi, Florence, Italy

**Keywords:** Frontotemporal dementia, Genetics, Language, Tau, Progranulin, *C9orf72*

## Abstract

**Background:**

Behavioural variant fronto-temporal dementia (bvFTD) is characterised by a progressive change in personality in association with atrophy of the frontal and temporal lobes. Whilst language impairment has been described in people with bvFTD, little is currently known about the extent or type of linguistic difficulties that occur, particularly in the genetic forms.

**Methods:**

Participants with genetic bvFTD along with healthy controls were recruited from the international multicentre Genetic FTD Initiative (GENFI). Linguistic symptoms were assessed using items from the Progressive Aphasia Severity Scale (PASS). Additionally, participants undertook the Boston Naming Test (BNT), modified Camel and Cactus Test (mCCT) and a category fluency test. Participants underwent a 3T volumetric T1-weighted MRI, with language network regional brain volumes measured and compared between the genetic groups and controls.

**Results:**

76% of the genetic bvFTD cohort had impairment in at least one language symptom: 83% *C9orf72*, 80% *MAPT* and 56% *GRN* mutation carriers. All three genetic groups had significantly impaired functional communication, decreased fluency, and impaired sentence comprehension. *C9orf72* mutation carriers also had significantly impaired articulation and word retrieval as well as dysgraphia whilst the *MAPT* mutation group also had impaired word retrieval and single word comprehension. All three groups had difficulties with naming, semantic knowledge and verbal fluency. Atrophy in key left perisylvian language regions differed between the groups, with generalised involvement in the *C9orf72* group and more focal temporal and insula involvement in the other groups. Correlates of language symptoms and test scores also differed between the groups.

**Conclusions:**

Language deficits exist in a substantial proportion of people with familial bvFTD across all three genetic groups. Significant atrophy is seen in the dominant perisylvian language areas and correlates with language impairments within each of the genetic groups. Improved understanding of the language phenotype in the main genetic bvFTD subtypes will be helpful in future studies, particularly in clinical trials where accurate stratification and monitoring of disease progression is required.

**Supplementary Information:**

The online version contains supplementary material available at 10.1007/s00415-022-11512-1.

## Background

Frontotemporal dementia (FTD) is a neurodegenerative disorder affecting particularly those under the age of 65 [1]. The most common presentation is the behavioural variant (bvFTD) [2], which is characterised by a progressive change in personality including loss of inhibitory control, apathy, obsessive–compulsive behaviour, reduced empathy and altered dietary preferences [3]. The main cognitive domains affected in bvFTD are executive function and social cognition [3, 4]. However, impairment in other domains has been described including episodic memory [4, 5] and language [4, 6–8].

Language difficulties are the presenting symptoms of the primary progressive aphasias (PPA), another form of FTD, of which there are three main subtypes: nonfluent variant (nfvPPA), where there are difficulties with grammar and/or speech apraxia, semantic variant (svPPA), where there is impaired naming and word comprehension, and logopenic variant (lvPPA), in which there are word retrieval problems [4, 9]. Of note, a number of patients do not fit criteria for any of the three core syndromes, often called PPA-unclassified, atypical PPA, or PPA-not otherwise specified (PPA-NOS) [10–12]**.** Features of each of the language variants have been described in people with bvFTD, but few studies have been performed to investigate the type or extent of linguistic impairment in bvFTD, and little is known about whether there are particular clinico-pathological associations for any of the symptoms [8, 13, 14].

Around a third of individuals with FTD have an autosomal dominant inheritance [15], with mutations in progranulin (*GRN*), microtubule-associated protein tau (*MAPT*) and chromosome 9 open reading frame 72 (*C9orf72*) being the most common causes [16]. BvFTD is the most frequent phenotype in all of the genetic forms of FTD with only a minority having PPA. However, like in sporadic disease, language problems have been described in people with genetic bvFTD [7, 17], albeit with little known so far about the exact features and whether differences exist between individuals within the main genetic groups (*GRN*, *MAPT* and *C9orf72*). Such information is important, not only to understand the disease better during the symptomatic period of the disorder, but also to further our knowledge of what symptoms to expect (and measure) in the prodromal stage of FTD. This would hopefully allow for improved stratification in clinical trials of disease-modifying treatments as well as more accurate measurement of treatment response.

This study therefore aims to explore the language phenotype of genetic bvFTD within the Genetic FTD Initiative (GENFI) cohort by investigating the linguistic features of the different genetic forms of FTD, including in relation to structural imaging measures.

## Methods

### Participants

Participants were recruited from the fifth data freeze of the GENFI study between 20 January 2012 and 30 May 2019, including sites in the UK, Canada, Belgium, France, Germany, Italy, the Netherlands, Portugal, Spain and Sweden. All aspects of the study were approved by local ethics committees, and written informed consent was obtained from all participants.

Participants underwent a standardised clinical assessment including a clinical history and neurological examination, neuro-psychometric assessment, the Mini-Mental State Examination (MMSE), and the CDR^®^ plus NACC FTLD [18]. The CDR^®^ plus NACC FTLD was used to classify mutation carriers as asymptomatic (global score of 0), prodromal (score 0.5) or symptomatic (score ≥ 1). To investigate the features of bvFTD, we reviewed all symptomatic mutation carriers recruited in the study and excluded those severely affected (CDR^®^ plus NACC FTLD score of 3 i.e. only included those with a score of 1 or 2). In total, 43 participants met consensus diagnostic criteria for bvFTD [3]: 24 with *C9orf72* expansions, 9 with *GRN* mutations and 10 with *MAPT* mutations. A comparison group of 100 healthy controls from the GENFI cohort (i.e. family members who did not carry a genetic mutation) was included, matched with the overall bvFTD group on age, sex, and years of education. Demographics are shown in Table 1.

**Table 1:** Demographics, clinical scores, severity of linguistic symptoms, cognitive task data and regional brain volumes for the bvFTD groups and healthy controls

	Controls	bvFTD		
		*C9orf72*	*GRN*	*MAPT*
Number of participants	100	24	9	10
% Male	45	67	44	**80**
% Right-handed	95	96	100	70
Age (years)	60.2 (7.1)	62.3 (7.9)	**67.2 (7.4)**	59.1 (7.7)
Education (years)	13.5 (3.1)	14.5 (3.6)	12.0 (3.2)	13.4 (3.5)
MMSE	29.1 (1.2)	**26.0 (3.4)**	**23.9 (5.1)**	**24.4 (5.0)**
CDR^®^ plus NACC FTLD Global score	0.1 (0.2)	**1.6 (0.5)**	**1.7 (0.5)**	**1.8 (0.4)**
CDR^®^ plus NACC FTLD Sum of Boxes	0.3 (0.6)	**8.5 (3.9)**	**8.0 (3.5)**	**9.0 (3.1)**
Progressive Aphasia Severity Scale Sum of Boxes	0.1 (0.4)	**2.7 (2.6)**	**2.2 (2.5)**	**3.3 (2.6)**
*Linguistic symptoms*				
Impaired articulation	0.07 (0.18)	**0.27 (0.53)**	0.06 (0.17)	0.10 (0.21)
Decreased fluency	0.00 (0.00)	**0.44 (0.61)**	**0.39 (0.36)**	**0.55 (0.50)**
Impaired grammar/syntax	0.00 (0.00)	0.06 (0.17)	0.11 (0.33)	0.15 (0.34)
Impaired word retrieval	0.08 (0.21)	**0.48 (0.56)**	0.44 (0.68)	**0.80 (0.59)**
Impaired speech repetition	0.01 (0.05)	0.04 (0.14)	0.06 (0.17)	0.00 (0.00)
Impaired sentence comprehension	0.00 (0.00)	**0.38 (0.61)**	**0.22 (0.36)**	**0.45 (0.69)**
Impaired single word comprehension	0.01 (0.05)	0.06 (0.17)	0.00 (0.00)	0.40 (0.66)^ab^
Dyslexia	0.02 (0.12)	0.08 (0.41)	0.11 (0.33)	0.15 (0.24)
Dysgraphia	0.01 (0.05)	**0.17 (0.32)**	0.22 (0.44)	0.15 (0.34)
Impaired functional communication	0.03 (0.13)	**0.73 (0.83)**	**0.56 (0.73)**	**0.55 (0.44)**
*Cognitive tasks*				
Boston Naming Test (/30)	27.8 (2.0)	**23.9 (4.0)**	24.3 (4.9)	**17.0 (7.7)**^**ab**^
Modified Camel and Cactus Test (/32)	30.0 (1.5)	**24.7 (5.2)**	**25.1 (4.3)**	**24.5 (5.9)**
Category Fluency (max in 60 s)	23.3 (5.6)	**13.0 (6.2)**	**12.4 (6.6)**	**12.7 (5.2)**
*Regional left hemisphere brain volumes (as a % of TIV)*				
Inferior frontal gyrus	0.56 (0.06)	**0.47 (0.09)**^**c**^	0.47 (0.08)	0.55 (0.06)
Insula	0.36 (0.03)	**0.28 (0.04)**	**0.30 (0.02)**	**0.26 (0.05)**^**b**^
Motor cortex	1.36 (0.12)	**1.21 (0.13)**^**c**^	**1.22 (0.07)**	1.33 (0.12)
Temporal pole	0.50 (0.06)	**0.43 (0.08)**	0.46 (0.07)	**0.35 (0.09)**^**ab**^
Superior temporal gyrus	0.48 (0.05)	**0.41 (0.04)**	**0.42 (0.05)**	**0.41 (0.04)**
Supratemporal region	0.40 (0.04)	**0.37 (0.04)**	**0.35 (0.05)**	**0.37 (0.04)**
Angular gyrus	0.50 (0.06)	**0.43 (0.05)**^**c**^	0.49 (0.08)	0.50 (0.08)

Data are shown as mean (standard deviation). Bold items are significantly different to controls

*bvFTD* behavioural variant fronto-temporal dementia; *TIV* total intracranial volume

^a^significantly impaired compared to *C9orf72* mutation carriers

^b^significantly impaired compared to *GRN* mutation carriers

^c^significantly impaired compared to *MAPT* mutation carriers

### Language symptom assessment

Language was assessed by a clinician using the GENFI linguistic symptom scale, which is based on the Progressive Aphasia Severity Scale (PASS) [19]. This contains ten language symptoms scored as per a clinical dementia rating scale i.e. 0 = asymptomatic, 0.5 = questionable/very mild, 1 = mild, 2 = moderate and 3 = severe: impaired articulation, decreased fluency, impaired grammar/syntax, impaired word retrieval, impaired speech repetition, impaired sentence comprehension, impaired single word comprehension, dyslexia, dysgraphia, and impaired functional communication.

### Linguistic and non-linguistic cognitive assessment

Within the GENFI neuropsychology battery, the 30-item version of the Boston Naming Test [20, 21] (BNT), the modified Camel and Cactus Test [22] (mCCT) and category fluency (animals) were the linguistic measures used.

The rest of the GENFI neuropsychology battery includes tests of attention and executive function including the Trail Making Test parts A and B (TMTA and TMTB), D-KEFS Colour-Word Inference Test (CWIT), WAIS-R Digit Symbol test, and WMS-R Digit Span Forwards (DSF) and Backward (DSB) as well as tests of visuospatial skills (WASI Block Design), episodic memory (the Free and Cued Selective Reminding Test, FCSRT) and social cognition (mini-Social Cognition and Emotion Assessment, mini-SEA, which includes a Faux Pas test of theory of mind and a Facial Emotion Recognition Test).

### Imaging

One hundred and twenty nine participants had a 3T volumetric T1-weighted magnetic resonance imaging (MRI) scan (46 Siemens Prisma, 18 Siemens Trio, 17 Siemens Skyra, 47 Philips Achieva, 1 GE Signa HD) of sufficient quality to be analysed: 37 patients with bvFTD (20 with *C9orf72*, 8 with *GRN*, and 9 with *MAPT* mutations) and 92 controls. Those without scans had either not been scanned due to contraindications or had a poor quality scan due to movement or other artefacts.

**Fig. 1:**
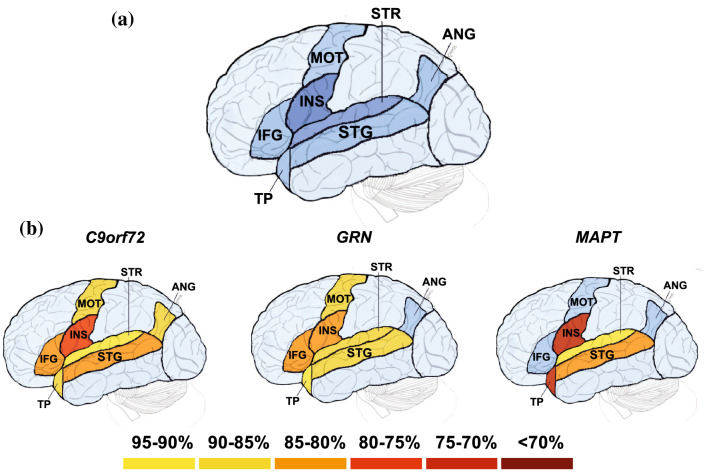
**a** Left perisylvian regions included in the MR imaging analysis are shown in this artificial representation of the lateral surface of the brain, with the insula and supratemporal region shown in darker blue to represent that they are deeper structures within the sylvian fissure, and **b** region of interest volumes in each genetic group as a percentage of mean control volume: *IFG* inferior frontal gyrus; *INS* insula; *MOT* motor cortex; *TP* temporal pole; *STG* superior temporal gyrus; *STR* supratemporal region; *ANG* angular gyrus. The darkest colours represent areas of lowest brain volume as per the key

Volumetric MRI scans were first bias field-corrected and whole brain parcellated using the geodesic information flow (GIF) algorithm [23], which is based on atlas propagation and label fusion. We focussed on key language regions that were present in the GIF parcellation atlas, calculating grey matter volumes of the cortex for seven left hemisphere perisylvian regions (Fig. 1a): inferior frontal gyrus, insula, motor cortex, temporal pole, superior temporal gyrus, supratemporal region, and angular gyrus. All measures were expressed as a percentage of total intracranial volume (TIV) computed with SPM12 v6470 (Statistical Parametric Mapping, Wellcome Trust Centre for Neuroimaging, London, UK) running under Matlab R2014b (Math Works, Natick, MA, USA) [24].

### Statistical analysis

All statistical analyses were performed using Stata/MP 16.1. Statistical tests of normality were performed using the Shapiro–Wilk test. Demographics were compared between groups using either linear regression (age and education) or a chi-squared test (sex). Linear regressions adjusting for age and sex were used to compare the MMSE, CDR^®^ plus NACC FTLD and PASS scores as well as the cognitive tasks and regional brain volumes between groups. Individual linguistic symptoms were compared in each disease group versus controls using linear regressions adjusting for age and sex, and 95% bias-corrected bootstrapped confidence intervals with 2000 repetitions (as there was minimal variation from zero in severity scores for the control group), and between genetic groups using an ordinal logistic regression adjusting for age and sex. Comparison between language-associated brain regions and both individual language symptoms and linguistic tasks was performed using Spearman rank correlations.

## Results

### Demographics

No significant differences were seen between the groups in years of education, but the *GRN* mutation carriers were significantly older than controls (*p* = 0.007) and *MAPT* mutation carriers (*p* = 0.020), and the *MAPT* mutation group had more males than controls (Chi^2^ = 4.46, *p* = 0.035) (Table 1).

### Disease severity

The MMSE and CDR^®^ plus NACC FTLD Sum of Boxes scores were significantly different to controls in each genetic group, but there were no significant differences between the genetic groups (Table 1).

### Language symptoms

**Fig. 2:**
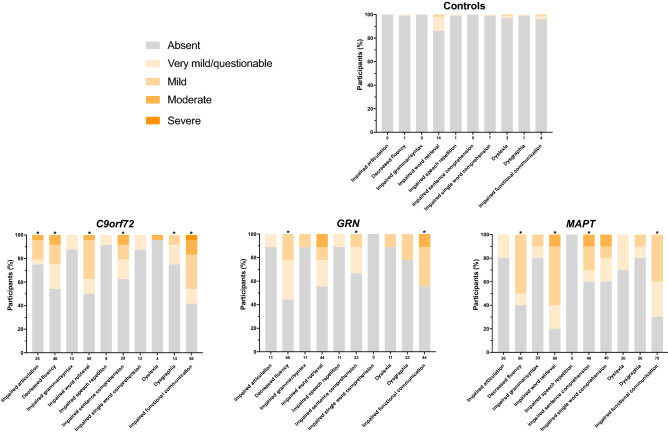
The percentage of participants in each of the groups who score 0 = absent, 0.5 = very mild/questionable, 1 = mild, 2 = moderate, or 3 = severe for each linguistic symptom. Values along the x-axis represent the frequency (%) with which the symptom is present in any severity category (0.5–3). An asterisk above the bar indicates that the symptom severity is significantly greater than controls

**Fig. 3:**
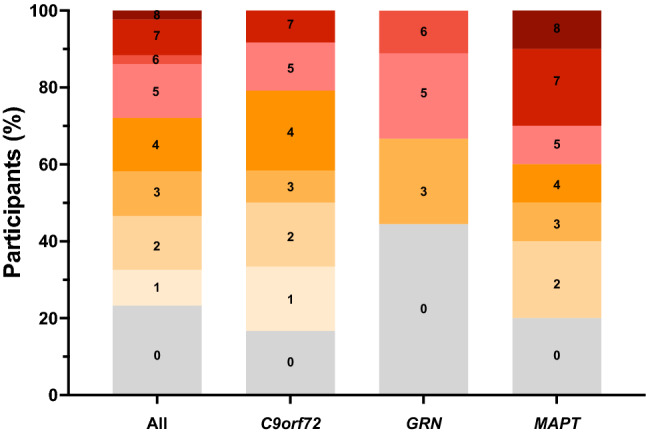
The percentage of participants with a particular number of language symptoms present, including all mutation carriers and each of the mutation groups

76% of the total bvFTD cohort had impairment in at least one language symptom (33 out of 43 participants): 83% of the *C9orf72* group, 56% of the *GRN* group and 80% of the *MAPT* group. In comparison, only 17% of the controls showed any impairment (Table 1, Fig. 2). However, a significant number of patients in the *MAPT* and *C9orf72* groups only had only one or two language symptoms, with a similar number of cases showing 3 or more language symptoms across all three groups (Fig. 3).

All three groups had significantly impaired functional communication compared to controls: severity mean 0.73 (standard deviation 0.83), frequency 58% in the *C9orf72* expansion carriers, 0.56 (0.73), 44% in the *GRN* mutation carriers and 0.55 (0.44), 70% in the *MAPT* mutation carriers. Similarly, all groups had significantly decreased fluency (0.44 (0.61), 46% in the *C9orf72* group, 0.39 (0.36), 56% in the *GRN* group and 0.55 (0.50), 60% in the *MAPT* group) and impaired sentence comprehension (0.38 (0.61), 29% in the *C9orf72* group, 0.22 (0.36), 33% in the *GRN* group and 0.45 (0.69), 40% in the *MAPT* group) compared to controls.

However, the pattern of linguistic symptomatology otherwise varied across the genetic groups. For the *C9orf72* group, impaired word retrieval (0.48 (0.56), 50%), impaired articulation (0.27 (0.53), 25%) and dysgraphia (0.17 (0.32), 14%) were significantly different to controls, whilst for the *MAPT* group, impaired word retrieval (0.80 (0.59), 80%) was significantly different to controls, and impaired single word comprehension (0.40 (0.66), 40%) was significantly different to both the other two genetic groups. No other symptoms were significantly different to controls or the other groups in the *GRN* mutation carriers.

### Cognitive assessment

The *C9orf72* and *MAPT* groups scored significantly lower than controls on the BNT (*p* < 0.001, Table 1), with a trend to a lower score in the *GRN* group (*p* = 0.060). The *MAPT* mutation carriers scored the lowest of the three groups, significantly lower than the *C9orf72* and *GRN* mutation carrier groups (*p* = 0.006 and *p* = 0.008 respectively). All three groups scored significantly lower than controls on the mCCT (*C9orf72*, *p* < 0.001; *GRN*, *p* = 0.005, *MAPT*, *p* = 0.009), and on category fluency (*p* < 0.001 for all three groups).

As expected, in the other cognitive tasks, all three genetic bvFTD groups showed evidence of executive dysfunction and impaired social cognition (Supplementary Table 1, Additional File 1). Additionally, significantly lower scores on the FCSRT and Block Design were seen in all three groups compared with controls, with DSF also significantly impaired in the *C9orf72* group (Supplementary Table 1, Additional File 1).

### Imaging analysis

All the left hemisphere regional brain volumes were significantly reduced compared to controls in the *C9orf72* expansion carriers (Table 1, Fig. 1b): insula (78% of mean control volume, *p* < 0.001), inferior frontal gyrus (84%, *p* = 0.002), superior temporal gyrus (85%, *p* < 0.001), temporal pole (86%, *p* < 0.001), angular gyrus (86%, *p* < 0.001), motor cortex (89%, *p* < 0.001) and supratemporal region (93%, *p* = 0.005). Inferior frontal gyrus, motor cortex and angular gyrus were significantly lower in volume compared to *MAPT* mutation carriers (*p* = 0.027, 0.012, and 0.005 respectively).

The *GRN* group had significantly reduced volume compared to controls in the insula (83%, *p* < 0.001), superior temporal gyrus (88%, *p* = 0.005), supratemporal region (88%, *p* = 0.041), and motor cortex (90%, *p* = 0.003), with a trend to a decreased volume in the inferior frontal gyrus (84%, *p* = 0.051) (Table 1, Fig. 1b).

The temporal pole was the most atrophied region in the *MAPT* mutation carriers (70%, *p* < 0.001) significantly lower than controls and the other mutation groups. The insula (72%, *p* < 0.001), superior temporal gyrus (85%, *p* < 0.001) and supratemporal region (93%, *p* = 0.043) were also significantly lower in volume compared with controls (Table 1, Fig. 1b).

For the linguistic symptoms, in the *C9orf72* group, impaired articulation significantly negatively correlated with volume of the inferior frontal gyrus (*r* = − 0.64, *p* = 0.002) and motor cortex (*r* = − 0.59, *p* = 0.008) whilst decreased fluency also negatively correlated with motor cortex volume (*r* = − 0.65, *p* = 0.002) as well as insula volume (*r* = − 0.54, *p* = 0.016) (Supplementary Table 2, Additional File 2). Impaired word retrieval negatively correlated with motor cortex volume (*r* = − 0.51, *p* = 0.025), and impaired functional communication negatively correlated with insula volume (*r* = − 0.54, *p* = 0.009). In the *GRN* group, impaired sentence comprehension negatively correlated with the volume of temporal regions: temporal pole (*r* = − 0.73, *p* = 0.040); superior temporal gyrus (*r* = − 0.78, *p* = 0.021), and supratemporal region (*r* = − 0.73, *p* = 0.040). Finally, in the *MAPT* group, decreased fluency (*r* = − 0.87, *p* = 0.012), impaired single word comprehension (*r* = − 0.79, *p* = 0.034), and dyslexia (*r* = − 0.79, *p* = 0.034) all negatively correlated with inferior frontal gyrus volume, whilst impaired sentence comprehension negatively correlated with angular gyrus volume (*r* = − 0.80, *p* = 0.030).

In the *C9orf72* group, there was a strong positive correlation between scores on the linguistic cognitive tasks and left inferior frontal gyrus, left insula and left angular gyrus volumes (Table 2): BNT (inferior frontal gyrus *r* = 0.50, *p* = 0.029; angular gyrus *r* = 0.77, *p* =  < 0.001); mCCT (inferior frontal gyrus *r* = 0.47, *p* = 0.044; insula *r* = 0.61, *p* = 0.005; angular gyrus *r* = 0.47, *p* = 0.043); CF (inferior frontal gyrus *r* = 0.67, *p* = 0.002; insula *r* = 0.50, *p* = 0.029). In the *GRN* group, there was a significant positive correlation between BNT score and temporal volumes: superior temporal gyrus *r* = 0.82, *p* = 0.013; supratemporal region *r* = 0.80, *p* = 0.018. Although there were no significant correlations of mCCT score with volumes, CF score was correlated with left insula and angular gyrus volumes (*r* = 0.86, *p* = 0.007; *r* = 0.74, *p* = 0.038 respectively). In *MAPT* mutation carriers, a strong positive correlation was seen between mCCT score and left superior temporal gyrus volume (*r* = 0.93, *p* = 0.003), but no significant correlations were seen for the BNT or CF scores.

**Table 2:** Correlations between the linguistic cognitive tasks and left hemisphere regional brain volumes in each genetic group

		BNT	mCCT	CF
Inferior frontal gyrus	*C9orf72*	**0.50**	**0.47**	**0.67**
	*GRN*	− 0.19	0.00	0.58
	*MAPT*	− 0.14	− 0.11	0.16
Insula	*C9orf72*	0.35	**0.61**	**0.50**
	*GRN*	0.16	0.21	**0.86**
	*MAPT*	0.63	0.48	0.34
Motor cortex	*C9orf72*	0.12	0.40	0.37
	*GRN*	0.35	0.31	0.45
	*MAPT*	− 0.34	− 0.07	0.41
Temporal pole	*C9orf72*	0.06	0.05	− 0.20
	*GRN*	0.43	0.38	0.46
	*MAPT*	0.41	0.15	− 0.20
Superior temporal gyrus	*C9orf72*	0.08	0.33	0.11
	*GRN*	**0.82**	0.48	0.17
	*MAPT*	0.45	**0.93**	0.56
Supratemporal region	*C9orf72*	0.26	0.14	0.17
	*GRN*	**0.80**	0.14	− 0.02
	*MAPT*	0.67	0.56	0.23
Angular gyrus	*C9orf72*	**0.77**	**0.47**	0.36
	*GRN*	0.06	0.21	**0.74**
	*MAPT*	− 0.74	− 0.26	− 0.05

Rho is shown for each correlation, with significant values shown in bold

*BNT* Boston naming test; *mCCT* modified Camel and Cactus Test; *CF* category fluency

## Discussion

In this study, we have shown that language impairment is present in a substantial proportion of people with the genetic form of bvFTD. Furthermore, significant atrophy is seen in the dominant perisylvian language regions and correlates with linguistic features in each of the three genetic groups. Three symptoms were seen significantly more than controls in all three genetic groups: impaired functional communication, decreased fluency and impaired sentence comprehension. Each group also had problems with naming, semantic knowledge and verbal fluency. However, differences were also present, with the *MAPT* mutation group having difficulties with single word comprehension and the *C9orf72* group having impaired articulation and dysgraphia. Similarly, whilst the three genetic groups had overlapping left insula atrophy, there were differences between them, with inferior frontal involvement particularly in the *C9orf72* group, anterior temporal atrophy in *MAPT* mutation carriers, and superior temporal volume loss in the *GRN* mutation carriers.

All three groups had difficulties with impaired functional communication. Whilst this could be in part due to individual or combined linguistic deficits, previous studies have shown that people with bvFTD can be impaired in conversational discourse and the pragmatics of language [25, 26]. People with bvFTD can have difficulties with participating in communication in the first place [26], as well as how best to communicate effectively [25]. These deficits are likely to arise from a combination of executive function and social cognition deficits that are present here in each of the three genetic groups [27, 28].

Similarly, impaired sentence comprehension was seen in all three groups. Difficulties in this domain can be due to abnormal grammatical processing, but this was not significantly impaired in any of the groups within this cohort. Problems in sentence comprehension can also be due to the inability to hold the sentence in short-term memory, as is seen in people with lvPPA, with shorter sentences easier to comprehend than longer ones [29]. The *C9orf72* mutation carriers had significantly shorter forwards digit span than controls (with a trend in *GRN* mutation carriers), but whilst this may have contributed to impaired sentence comprehension, this deficit in short-term memory was not as severe as that seen in lvPPA. Lastly, separate to grammar and short-term memory, prior research in FTD has suggested a role for decreased executive resources as a contributor to impaired sentence comprehension [30, 31], and certainly all three groups had significant executive dysfunction on testing here. It may well be therefore that a combination of executive dysfunction and impaired short-term memory leads to impaired sentence comprehension across the different groups.

Also seen in all three groups was decreased fluency. This can be caused by multiple underlying linguistic deficits including changes in articulation, grammar and word retrieval as well as an impairment of word generation or prosody [32]. The *C9orf72* mutation group had significant articulatory deficits and both *C9orf72* and *MAPT* mutation groups had word retrieval problems, but all three groups also had decreased verbal fluency scores compared to controls. Word generation difficulties are a major contributor to decreased verbal fluency scores in bvFTD [33], with associated executive dysfunction being an important cause [34]. Prosodic changes have also previously been noted in bvFTD with changes in different aspects of speech timing found in one study [32]. It is therefore likely that the decreased fluency noted on the linguistic symptom scale here is multifactorial in each group with contributions from different factors.

The *C9orf72* mutation carriers had the most language impairment of any of the groups, with 83% having deficits in at least one symptom. As well as the difficulties above, 25% of patients were noted to have impaired articulation. It was not noted if this was due to apraxia of speech, dysarthria or other reasons in the symptom scales, but the score did correlate with atrophy in the inferior frontal gyrus and motor cortex. None of these carriers had an associated diagnosis of amyotrophic lateral sclerosis (ALS), but prior studies have shown the presence of motor deficits in people with FTD that do not meet criteria for ALS [35]. Correlation with inferior frontal cortex volume and/or the insula was seen also for decreased fluency and performance on the cognitive language tasks, suggesting that the impairments are not just down to motor speech deficits, but that there are true language-based speech production difficulties as well. The *C9orf72* group had more widespread involvement of the perisylvian language areas in general, including more posterior regions like the angular gyrus. Interestingly, scores on the BNT and mCCT both correlated significantly with angular gyrus atrophy. This region forms part of what has been called Geschwind’s area and is associated with multiple language functions [36] including word retrieval and semantic processing as well as writing, which is also impaired in the *C9orf72* mutation carrier group. It is therefore likely that the more extensive linguistic deficits seen in this group relate to the wider involvement of key linguistic regions in the brain as the disease progresses in addition to a contribution from executive dysfunction. As mentioned above, this latter cognitive deficit is likely to be a factor in a number of apparent language problems [34], including the decreased score in the mCCT, which has previously been shown to be related to the executive control of semantic knowledge [22].

80% of the *MAPT* mutation group had linguistic symptoms with single word comprehension being impaired more than the other two groups. The *MAPT* group also included individuals with the greatest number of language symptoms affected, and with the lowest naming scores of all three groups. The group also had impairments in the mCCT and in verbal fluency. Previous studies of *MAPT-*associated bvFTD have highlighted the presence of semantic impairment, a feature that can occur quite early in the disease process, even prodromally [22, 37, 38]. This is generally associated with atrophy of the anterior temporal lobes (the ‘semantic hub’), as is seen prominently here, with mCCT score correlating with temporal lobe atrophy. Interestingly, there was also a correlation of decreased fluency, impaired single word comprehension and dyslexia with inferior frontal gyrus volume in this group, suggesting more wider contributions to the language deficits.

The *GRN*-bvFTD had the least linguistic symptomatology of all the groups. This sits in contrast to *GRN* mutations being the commonest cause of PPA in genetic FTD, with usually either a nonfluent variant or a mixed (not otherwise specified) phenotype [12, 39]. It may be that many of the linguistic deficits seen here are related to the executive dysfunction found in the group, but in fact impaired sentence comprehension as well as BNT score correlated with temporal lobe volumes, suggesting a primary linguistic impairment, potentially of semantics within the *GRN* group.

An important question arises about what diagnosis should be given to people who have bvFTD but also early, prominent language deficits. The current diagnostic criteria are poorly equipped for situations like this e.g. to fulfil a diagnosis of PPA the ‘most prominent clinical feature’ must be ‘difficulty with language’ i.e. there is no current option to make a dual diagnosis of bvFTD with PPA (or even ‘secondary’ progressive aphasia). Future revisions of the diagnostic criteria should consider this issue.

### Limitations

In general, despite a well-defined cohort in the GENFI study, once groups were stratified, the numbers that could be studied were much smaller. Further studies of larger genetic FTD cohorts to replicate these findings will be helpful. Another limitation was the limited availability of language cognitive tests within the GENFI battery. There is a lack of validated cross-language verbal linguistic tasks in general and, with multiple languages represented in the GENFI study, the cognitive battery has mostly non-verbal or already validated tasks. Future studies should therefore develop novel cross-language tasks with normative data that can be used in multinational studies like GENFI.

### Conclusions

Despite a primary bvFTD diagnosis, language problems are present extensively in genetic FTD, with overlapping but distinct patterns of linguistic deficits and associated brain atrophy. Improved understanding of the relationship between bvFTD and its language phenotype will aid more focussed assessments and interpretations of data within FTD studies. This in turn will guide the future stratification of individuals within clinical trials as well as the monitoring of disease progression and treatment response.

## Supplementary Information

Below is the link to the electronic supplementary material.

Supplementary file1 (PDF 140 KB)

## Data Availability

The datasets used and/or analysed during the current study are available from the corresponding author on reasonable request.

## Abbreviations

ALS: Amyotrophic lateral sclerosis
bvFTD: Behavioural variant fronto-temporal dementia
BNT: Boston naming test
*C9orf72*: Chromosome 9 open reading frame 72
CWIT: D-KEFS colour-word interference test
FCSRT: Free and cued selective reminding test
FTD: Frontotemporal dementia
GENFI: Genetic frontotemporal dementia initiative
GIF: Geodesic information flow
lvPPA: Logopenic variant primary progressive aphasia
MRI: Magnetic resonance imaging
*MAPT*: Microtubule-associated protein tau
MMSE: Mini-mental state examination
mini-SEA: Mini-social cognition and emotion assessment
mCCT: Modified camel and cactus test
nfvPPA: Non-fluent variant primary progressive aphasia
PPA: Primary progressive aphasia
*GRN*: Progranulin
PASS: Progressive aphasia severity scale
svPPA: Semantic variant primary progressive aphasia
TIV: Total intracranial volume
TMT: Trail making test
DSF/DSB: WMS-R digit span forward/backward
