# Supplementary material for: Language impairment in the genetic forms of behavioural variant frontotemporal dementia
Source: J Neurol. 2022 Dec 20;270(4):1976–88. doi: 10.1007/s00415-022-11512-1 (PMC10025186; doi:10.1007/s00415-022-11512-1)
Supplement: Supplementary file 1 — Supplementary file1 (PDF 140 KB) [file 415_2022_11512_MOESM1_ESM.pdf]

Language impairment in the genetic forms of behavioural variant frontotemporal dementia

Kiran Samra[1], Amy M. MacDougall[2], Arabella Bouzigues[1], Martina Bocchetta[1], David M. Cash[1], Caroline V. Greaves[1], Rhian S. Convery[1], John C. van Swieten[3], Harro Seelaar[3], Lize Jiskoot[3], Fermin Moreno[4,5], Raquel Sanchez-Valle[6], Robert Laforce[7], Caroline Graff[8,9], Mario Masellis[10], Maria Carmela Tartaglia[11], James B. Rowe[12], Barbara Borroni[13], Elizabeth Finger[14], Matthis Synofzik[15,16], Daniela Galimberti[17,18], Rik Vandenberghe[19,20,21], Alexandre de Mendonça[22], Chris R Butler[23,24], Alexander Gerhard[25,26], Simon Ducharme[27,28], Isabelle Le Ber[29,30,31,32], Pietro Tiraboschi[33], Isabel Santana[34,35], Florence Pasquier[36,37,38], Johannes Levin[39,40,41], Markus Otto[42], Sandro Sorbi[43,44], Jonathan D. Rohrer[1*], Lucy L. Russell[1*], on behalf of the Genetic FTD Initiative (GENFI)[#]

*joint senior authors

[#]List of consortium authors in acknowledgements

[1]Dementia Research Centre, Department of Neurodegenerative Disease, UCL Queen Square Institute of Neurology, London, UK

[2]Department of Medical Statistics, London School of Hygiene and Tropical Medicine, London, UK

[3]Department of Neurology, Erasmus Medical Centre, Rotterdam, Netherlands

[4]Cognitive Disorders Unit, Department of Neurology, Donostia Universitary Hospital, San Sebastian, Spain

[5]Neuroscience Area, Biodonostia Health Research Institute, San Sebastian, Gipuzkoa, Spain

[6]Alzheimer's disease and Other Cognitive Disorders Unit, Neurology Service, Hospital Clínic, Institut d'Investigacións Biomèdiques August Pi I Sunyer, University of Barcelona, Barcelona, Spain

[7]Clinique Interdisciplinaire de Mémoire, Département des Sciences Neurologiques, CHU de Québec, and Faculté de Médecine, Université Laval, QC, Canada

[8]Center for Alzheimer Research, Division of Neurogeriatrics, Department of Neurobiology, Care Sciences and Society, Bioclinicum, Karolinska Institutet, Solna, Sweden

[9]Unit for Hereditary Dementias, Theme Aging, Karolinska University Hospital, Solna, Sweden

[10]Sunnybrook Health Sciences Centre, Sunnybrook Research Institute, University of Toronto, Toronto, Canada

[11]Tanz Centre for Research in Neurodegenerative Diseases, University of Toronto, Toronto, ON, Canada

[12]Department of Clinical Neurosciences, University of Cambridge, UK

[13]Neurology Unit, Department of Clinical and Experimental Sciences, University of Brescia, Brescia, Italy

[14]Department of Clinical Neurological Sciences, University of Western Ontario, London, ON, Canada

[15]Department of Neurodegenerative Diseases, Hertie-Institute for Clinical Brain Research and Center of Neurology, University of Tübingen, Tübingen, Germany

[16]Center for Neurodegenerative Diseases (DZNE), Tübingen, Germany

[17]Fondazione Ca' Granda, IRCCS Ospedale Policlinico, Milan, Italy

[18]University of Milan, Centro Dino Ferrari, Milan, Italy
[19]Laboratory for Cognitive Neurology, Department of Neurosciences, KU Leuven, Leuven, Belgium
[20]Neurology Service, University Hospitals Leuven, Leuven, Belgium
[21]Leuven Brain Institute, KU Leuven, Leuven, Belgium
[22]Laboratory of Neurosciences, Institute of Molecular Medicine, Faculty of Medicine, University of Lisbon, Lisbon, Portugal
[23]Nuffield Department of Clinical Neurosciences, Medical Sciences Division, University of Oxford, Oxford, UK
[24]Department of Brain Sciences, Imperial College London, UK
[25]Division of Neuroscience and Experimental Psychology, Wolfson Molecular Imaging Centre, University of Manchester, Manchester, UK
[26]Departments of Geriatric Medicine and Nuclear Medicine, University of Duisburg-Essen, Germany
[27]Department of Psychiatry, McGill University Health Centre, McGill University, Montreal, Québec, Canada
[28]McConnell Brain Imaging Centre, Montreal Neurological Institute, McGill University, Montreal, Québec, Canada
[29]Sorbonne Université, Paris Brain Institute – Institut du Cerveau – ICM, Inserm U1127, CNRS UMR 7225, AP-HP - Hôpital Pitié-Salpêtrière, Paris, France
[30]Centre de référence des démences rares ou précoces, IM2A, Département de Neurologie, AP-HP - Hôpital Pitié-Salpêtrière, Paris, France
[31]Département de Neurologie, AP-HP - Hôpital Pitié-Salpêtrière, Paris, France
[32]Reference Network for Rare Neurological Diseases (ERN-RND)
[33]Fondazione IRCCS Istituto Neurologico Carlo Besta, Milano, Italy
[34]University Hospital of Coimbra (HUC), Neurology Service, Faculty of Medicine, University of Coimbra, Coimbra, Portugal
[35]Center for Neuroscience and Cell Biology, Faculty of Medicine, University of Coimbra, Coimbra, Portugal
[36]Univ Lille, France
[37]Inserm 1172, Lille, France
[38]CHU, CNR-MAJ, Labex Distalz, LiCEND Lille, France
[39]Department of Neurology, Ludwig-Maximilians Universität München, Munich, Germany
[40]German Center for Neurodegenerative Diseases (DZNE), Munich, Germany
[41]Munich Cluster of Systems Neurology (SyNergy), Munich, Germany
[42]Department of Neurology, University of Ulm, Germany
[43]Department of Neurofarba, University of Florence, Italy
[44]IRCCS Fondazione Don Carlo Gnocchi, Florence, Italy

Corresponding author:

Dr Lucy L. Russell, Dementia Research Centre, Department of Neurodegenerative Disease, UCL Institute of Neurology, Queen Square, London, WC1N 3BG, l.russell@ucl.ac.uk

Online only (supplementary) material

Number of supplementary tables: 2

Number of supplementary figures: 0

Supplementary Table 1. Neuropsychology assessment scores for the bvFTD groups and controls. Bold items are significantly different to controls; [a]significantly impaired compared to *C9orf72* mutation carriers, [b]significantly impaired compared to *GRN* mutation carriers, [c]significantly impaired compared to *MAPT* mutation carriers.

Supplementary Table 2. Correlations between the linguistic symptoms and left hemisphere regional brain volumes in each genetic group. Rho is shown for each correlation, with significant values shown highlighted and in bold. Abbreviations: comp = comprehension.

Supplementary Table 1. Neuropsychology assessment scores for the bvFTD groups and controls. Bold items are significantly different to controls; [a]significantly impaired compared to *C9orf72* mutation carriers, [b]significantly impaired compared to *GRN* mutation carriers, [c]significantly impaired compared to *MAPT* mutation carriers.

	Controls		*C9orf72*		*GRN*		*MAPT*	
Trail Making Test Part A (max 150s)	30.8	(13.4)	**60.4**	**(31.8)**	**84.1**	**(41.2)[c]**	**45.1**	**(17.1)**
Trail Making Test Part B (max 300s)	73.5	(34.7)	**195.0**	**(89.3)**	**218.4**	**(112.8)**	**146.5**	**(88.3)**
D-KEFS Color-Word Interference Test (max 180s)	52.4	(13.1)	**110.0**	**(47.7)**	88.7	(50.9)	**79.5**	**(35.5)**
Digit Symbol test (max in 90s)	51.8	(12.3)	**27.8**	**(11.9)**	**22.6**	**(12.8)**	**33.2**	**(8.7)**
Digit Span Forwards (/12)	7.8	(1.9)	**6.3**	**(2.7)**	6.2	(2.7)	7.8	(2.6)
Digit Span Backwards (/12)	6.4	(2.0)	**4.3**	**(2.1)[c]**	**4.3**	**(2.8)**	6.5	(3.2)
Block Design (/71)	46.0	(14.2)	**18.8**	**(14.5)[c]**	**11.8**	**(9.8)[c]**	**37.7**	**(14.9)**
FCSRT - free recall (/48)	30.5	(7.1)	**16.7**	**(7.4)**	**6.7**	**(10.0)[a]**	**10.4**	**(7.3)[a]**
FCSRT - free + cued recall (/48)	45.3	(4.8)	**39.5**	**(8.5)**	**18.3**	**(16.4)[a]**	**28.3**	**(12.4)[a]**
FCSRT - free delayed recall (/16)	11.4	(2.8)	**6.3**	**(3.8)**	**2.9**	**(4.3)**	**3.3**	**(4.2)**
FCSRT - free + cued delayed recall (/16)	15.2	(1.7)	**13.8**	**(2.6)**	**8.7**	**(7.0)**	**10.1**	**(4.5)[a]**
Mini-SEA: Faux-Pas test (/40)	34.7	(4.9)	**26.7**	**(7.5)**	**21.8**	**(5.6)[c]**	30.6	(7.3)
Mini-SEA: Facial Emotion Recognition Test (/35)	28.2	(3.3)	**20.9**	**(4.6)**	**17.1**	**(5.5)[c]**	**23.4**	**(6.0)**

Supplementary Table 2. Correlations between the linguistic symptoms and left hemisphere regional brain volumes in each genetic group. Rho is shown for each correlation, with significant values shown highlighted and in bold. Abbreviations: comp = comprehension.

		Impaired articulation	Decreased fluency	Impaired grammar	Impaired word retrieval	Impaired speech repetition	Impaired sentence comp	Impaired single word comp	Dyslexia	Dysgraphia	Impaired functional communication
**Inferior frontal gyrus**	***C9orf72***	**-0.64**	-0.26	0.21	-0.04	-0.39	0.14	0.16		-0.07	-0.11
	GRN	0.08	0.62	0.58	0.47	0.58	0.08		-0.25	0.25	0.20
	MAPT		**-0.87**		-0.56		-0.22	**-0.79**	**-0.79**	-0.45	-0.39
**Insula**	***C9orf72***	-0.45	**-0.54**	0.08	-0.39	-0.09	-0.07	0.21		0.46	**-0.58**
	GRN	-0.58	0.24	0.58	-0.17	0.58	-0.30		-0.25	0.25	-0.26
	MAPT		-0.58		-0.51		0.58	-0.16	-0.16	-0.40	-0.39
**Motor Cortex**	***C9orf72***	**-0.59**	**-0.65**	-0.29	**-0.51**	-0.30	-0.11	0.03		0.20	-0.26
	GRN	-0.41	0.24	-0.08	0.05	-0.08	-0.04		0.41	0.25	0.31
	MAPT		0.29		0.32		-0.22	0.63	0.63	-0.22	0.46
**Temporal pole**	***C9orf72***	-0.21	-0.36	-0.34	-0.38	0.26	0.05	0.34		0.30	0.01
	GRN	-0.58	-0.04	0.41	-0.13	0.41	**-0.73**		-0.41	0.00	-0.40
	MAPT		-0.14		0.09		0.76	0.16	0.16	-0.09	0.15
**Superior temporal gyrus**	***C9orf72***	-0.18	-0.42	-0.26	-0.27	0.09	-0.10	0.37		0.15	-0.13
	GRN	-0.41	0.01	0.58	0.13	0.58	**-0.78**		-0.25	0.25	-0.53
	MAPT		-0.14		-0.21		0.04	0.00	0.00	-0.45	-0.54
**Supratemporal region**	***C9orf72***	-0.40	-0.42	0.05	-0.12	-0.39	0.14	0.50		0.11	-0.42
	GRN	-0.08	0.03	0.58	0.33	0.58	**-0.73**		-0.41	0.25	0.31
	MAPT		-0.14		-0.09		0.76	0.32	0.32	-0.27	-0.08
**Angular gyrus**	***C9orf72***	-0.08	-0.03	0.08	0.37	0.30	0.40	0.24		0.13	-0.07
	GRN	-0.58	-0.11	0.41	-0.36	0.41	-0.54		-0.41	0.00	-0.50
	MAPT		0.29		0.30		**-0.80**	-0.16	-0.16	0.13	0.08
